# Treatment of Diseased Gums

**Published:** 1869-05

**Authors:** 


Treatment of Diseased Gums.—A writer in the *London Lancet* recommends the following treatment of diseased gums: The teeth should be washed night and morning with a moderately small and soft brush; after the morning ablution, pour on a second toothbrush, slightly damped, a little of the following lotion, and apply it to the affected parts: Carbolic acid, one scruple; rectified spirits of wine, two drachms; distilled water, six ounces. By the use of this preparation, suppurative action is kept under, and the gums get firmer and less tender.

